# Plasmon‐Driven Gold Nanopillar Multiarrayed Gene Amplification Methodology for the High‐Throughput Discrimination of Pathogens

**DOI:** 10.1002/advs.202411849

**Published:** 2025-01-14

**Authors:** Sung Eun Seo, Kyung Ho Kim, Seo Jin Kim, Kyong‐Cheol Ko, Woo‐Keun Kim, Kyoung G. Lee, Oh Seok Kwon

**Affiliations:** ^1^ SKKU Advanced Institute of Nanotechnology (SAINT) Sungkyunkwan University Suwon 16419 South Korea; ^2^ Korea Preclinical Evaluation Center Korea Research Institute of Bioscience and Biotechnology (KRIBB) 125 Gwahak‐ro, Yuseong‐gu Daejeon 34141 South Korea; ^3^ Department of Predictive Toxicology Korea Institute of Toxicology 141 Gajeong‐ro, Yuseong‐gu Daejeon 34114 South Korea; ^4^ Center for NanoBio Development National NanoFab Center (NNFC) Daejeon 34141 South Korea; ^5^ Department of Nano Science and Technology Sungkyunkwan University Suwon 16419 South Korea; ^6^ Department of Nano Engineering Sungkyunkwan University Suwon 16419 South Korea

**Keywords:** environmental monitoring, Gold nanopillar array, pathogen diagnosis, photothermal energy conversion, plasmonic PCR

## Abstract

Molecular diagnosis limitations, including complex treatment processes, low cost‐effectiveness, and operator‐dependent low reproducibility, interrupt the timely prevention of disease spread and the development of medical devices for home and outdoor uses. A newly fabricated gold nanopillar array‐based film is presented for superior photothermal energy conversion. Magnifying the metal film surface‐to‐volume ratio increases the photothermal energy conversion efficiency, resulting in a swift reduction in the gene amplification reaction time. Plasmonic energy‐based ultrafast gene amplification and facile confirmation methodology offer a rapid disease discrimination platform for high‐throughput multiplexed diagnosis. The superior performance of the gold nanopillar arrayed film is demonstrated by measuring the amount of pathogen (*Vibrio cholerae*) with a sensitivity of 10^1^ cfu mL^−1^ in 5.5 min. The newly engineered gold nanopillar arrayed film can be utilized to diagnose universal pathogens to achieve an increasingly successful complete cure.

## Graphical abstract

Those limitations of the present diagnosis platform interrupt the timely prevention of disease spread and the development of medical devices for facile usage. Herein, a novel molecular diagnosis methodology is presented with gold nanopillar array‐based film for superior photothermal energy conversion. The ultrastable metal surface interfacing chemistry (OPE) and plasmonic energy‐based ultrafast gene amplification methodology provides a rapid disease discrimination platform for high‐throughput diagnosis.

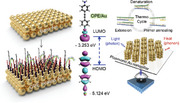

## 1 Introduction

The early‐stage diagnosis of disease and prescription of drugs for a successful complete cure have become emerging issues after pandemic outbreaks.^[^ ^1^ ^]^ Although molecular diagnosis such as polymerase chain reaction (PCR) is the gold standard, advanced technology is needed for the sensitive and high‐throughput analysis of pathogens.^[^ ^2^, ^3^ ^]^ Notably, the limitations of conventional PCR devices include the high costs of PCR kit solutions, the need for trained operators, and the large volume of the thermal energy generator (heating block).^[^ ^4^, ^5^, ^6^ ^]^ Based on the genomic amplification reaction, several trials have aimed to replace each component of the PCR device. While the conventional PCR device requires 20–25 µL per reaction, reconstructing the amplification chamber into nanosized wells enabled a decrease in the reaction solution volume to tens of nanoliters.^[^ ^7^, ^8^ ^]^ In conventional PCR, thermal energy is often generated via electrical current–thermal energy conversion.^[^ ^9^ ^]^ A large module is removed, and a set of LEDs and a light‐focusing lens are then located under the PCR chamber.^[^ ^10^ ^]^ Replacing the Peltier‐based heating module with a plasmonic‐based energy generator triggered a dramatic decrease in the entire device size. Furthermore, instead of the PCR tube, variously designed PCR chambers containing metal layers are utilized.^[^ ^11^, ^12^, ^13^ ^]^ Light is irradiated onto the metal layer deposited on the upper surface of the transparent substrate (glass, transparent polymer) for the generation of thermal energy.^[^ ^7^ ^]^ While the metal layer formation is developed for outperforming results, the structure varies from nano/microparticle, nanoarray/nanopillar, and hybrid structural nanomaterials.^[^ ^14^, ^15^, ^16^ ^]^ In previous research, the metal nanopillar array was utilized as the substrate for the detection of multiple biomarkers which enables the diagnosis of disease. However, the application was limited to surface‐enhanced Raman spectroscopy (SERS)^[^ ^17^ ^]^ and localized surface plasmon resonance (LSPR) as the intensity of the generated signal is very low.^[^ ^18^ ^]^ In this study, we present a plasmonic PCR device with an Au nanopillar arrayed chip to increase the gene amplification reaction efficiency. The uniformly arrayed Au nanostructure over a large area of glass demonstrated superior light‐to‐heat energy conversion due to its high surface‐to‐volume ratio.^[^ ^19^, ^20^, ^21^ ^]^ The whole amplification reaction was conducted for 5.5 min for 40 thermal cycles. Compared to a flat Au layer, the Au nanopillar arrayed substrate showed a rapid thermal cycle and efficient gene amplification results.^[^ ^7^ ^]^ As the light was irradiated to the nanopillar array loaded chip, photon energy was converted into thermal energy through the photon–electron–phonon conversion reaction.^[^ ^22^ ^]^ By maximizing the Au layer surface via various nanopillar patterns, more thermal energy was transferred to the PCR solution. In addition, the heated solution was cooled down rapidly with increased exposure of the metal surface to the PCR solution compared to the flat Au layer. Furthermore, the amplified results were easily confirmed using thermally–stable chemical linkers (OPEs). The OPE‐linked Au surface was treated with target‐specific primer. Amplicon production was observed through fluorescence emission from the Au surface. Intensifying the gene amplification yielded a higher intensity of fluorescence emitted from the Au nanopillar arrayed chip. In previous studies, thiol‐based and carbene compounds were frequently utilized for interfacial chemistry on metal surfaces (e.g., Au and silver).^[^ ^23^ ^]^ However, these interfacial chemistries presented fatal disadvantages: low thermal stability, complex synthesis processes, and low synthesis yields.^[^ ^24^ ^]^ In this study, we introduced a newly invented chemical linker (OPE) for primer conjugation onto the Au nanopillar surface. As presented in the density functional theory (DFT) simulation results, the binding energies of thiol and OPE were −423.5369 and −805.7225 Hartree, respectively, indicating that the OPE‐Au bond showed approximately two times higher binding energy than the thiol‐Au bond. Applying a novel organic interfacial compound enabled the direct confirmation of amplicon synthesis. During the gene amplification reaction in the chamber, the conjugated primer was also annealed to the single stranded‐DNA. The formation of double‐stranded DNA (dsDNA) was confirmed by observing the fluorescence emission produced by the chelated dye incorporated into the dsDNA amplified on the Au nanopillar surface. With the increased metal surface area due to the nanopillar structure, higher fluorescence emissions occurred from amplicon formation. As a result, the limit of detection (LOD) for this platform was lowered to 10^1^ cfu mL^−1^ using a DNA genomic sample of *Vibrio cholerae*. The results demonstrated that our newly developed interfacing chemistry, integrated with the plasmonic‐based Au nanopillar arrayed gene amplification methodology, allows for a highly efficient and rapid diagnosis of diverse pathogenic genomes, completing the total assay in just a few minutes.

## 2 Results and Discussion

### 2.1 Fabrication of Au Nanopillar Substrate

**Figure 1:**
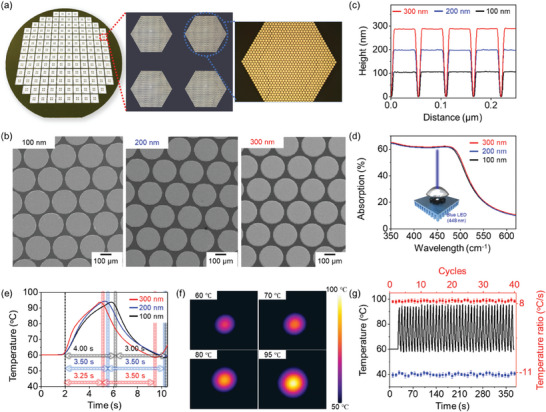
a) Photograph of the mass‐produced Au nanopillar onto an 8‐inch wafer and the specimen to show the separated area, and the construction of the specific area. b) Height‐dependent SEM images of the Au nanopillar. c) AFM profile corresponding to the SEM results. d) UV–vis spectra of the different pillar heights. e) Time profile for one photothermal cycle at different nanopillar heights. f) Thermal IR images based on temperature variance. g) Thermal profiles toward 45 thermal cycles (black line), heating (red scatter), and cooling velocities (blue scatter).

**Figure** **1**a depicts a photograph showing the mass‐produced chips for nanopillar solid‐phase photonic PCR on an 8‐inch wafer based on the MEMS process. The chips were fabricated with separated multi‐area nanopillar shapes by deposition, allowing for the conjugation of various primers depending on the targets (Figure 1a blue dots). The components were constructed using a honeycomb‐patterned Au nanopillar to enhance the fluid flow of the PCR solution in the chambers (Figure 1a red dots).^[^ ^12^ ^]^ Au nanopillars with 100–300 nm heights were fabricated to investigate the effects of light source absorption and photon‐thermal conversion (Figure 1b). Scanning electron microscope (SEM) images of Au nanopillar surface morphology revealed constant uniformity across varying nanopillar heights. Additionally, atomic force microscopy (AFM) measurements were performed to observe uniformity corresponding to the SEM images (Figure 1c). All Au nanopillar substrates exhibited a consistent height corresponding to each thickness, indicating an increase in the functionalizable surface area due to the enhanced uniformity of the surface.^[^ ^25^ ^]^ UV–vis measurements were conducted to investigate absorption based on the increased height of the Au nanopillar (Figure 1d). The absorption spectra displayed similar bands at the 350–650 nm wavelength; in particular, the absorption ratio showed distinction within 1%. Absorption based on Au nanopillar height exhibited no significant efficiency. Based on these results, a photon‐thermal conversion test was conducted to confirm the heating and cooling efficiency of the blue LED light source (450 nm) (Figure 1e). The honeycomb substrates, categorized by height, demonstrated similar rapid velocities within 12 s. The time required for one photothermal cycle in the temperature range of 60–95 °C was 7.00 s for 100 nm, 7.00 s for 200 nm, and 6.75 s for 300 nm substrates. Additionally, the heating/cooling ratios were 8.75/11.67 °C s^−1^ for 100 nm, 10.00/10.00 °C s^−1^ for 200 nm, and 10.77/10.00 °C s^−1^ for 300 nm. Among the various heights of Au nanopillar, the 100 nm was selected to consider the cost efficiency. To investigate the temperature variance of the Au nanopillar surface, thermal infrared camera images were obtained as the temperature changed using a thermal IR camera (Figure 1f). The temperature range of 60–95 °C was selected based on the reaction requirements of the general PCR kits. The results showed specific sections at 60, 70, 80, and 95 °C. Photothermal changes on the Au surface were observed from the center area, with the photothermal transfer spreading rapidly to all areas over time. Forty thermal cycles were conducted to confirm the consumption time and heating/cooling efficiency, with a total time requirement of 330 s (Figure 1g). The heating and cooling efficiencies were calculated at each cycle, and the average values were 8.75 and 11.67 °C s^−1^, respectively. In addition, the thermal deviation fell below 0.7 °C. These results indicate that honeycomb‐based PCR chip platforms can offer rapid and stable photothermal conversion in PCR reactions. Additionally, the structural stability before and after thermal exposure was carried out through SEM analysis, the nanopillar structure was confirmed by no significant change, and the stable nanostructure for thermal energy (Figure S1, Supporting Information).

### 2.2 Surface Treatment of Nanopillar Substrate and Analytical Results

**Figure 2:**
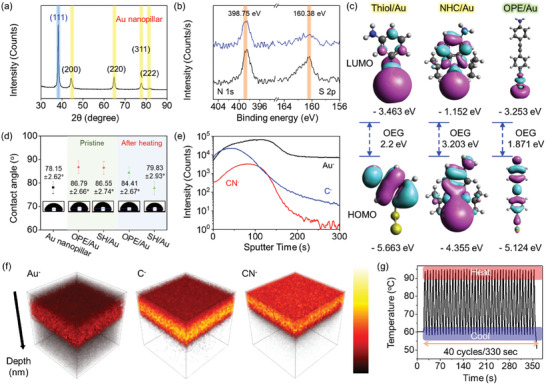
a) XRD spectrum to confirm the (111) plane of the Au nanopillar. b) XPS narrow spectra toward N 1s and S 2p core levels. c) The obtained 3D structures from the DFT simulation. d) Contact angle results of pristine Au nanopillar, OPE‐/SH‐introduced Au nanopillar, and heat‐treated OPE‐/SH‐introduced Au nanopillar. TOF‐SIMS e) profiles and f) 3D mapping images toward Au, C, and C‐N in the OPE‐introduced Au nanopillar. g) Thermocycle (60–95 °C) profile using an OPE‐introduced Au nanopillar.

A covalent bond is formed onto the Au nanopillar based on the radical reaction on the Au (111) plane. Surface analyses such as X‐ray diffraction (XRD), X‐ray photoelectron spectroscopy (XPS), contact angle measurements, and time‐of‐flight secondary ion mass spectrometry (TOF‐SIMS) were performed to investigate the covalent bonding between Au nanopillars and OPE. XRD surface analysis was performed to confirm the surface property of the Au nanopillar owing to the reaction of OPE with the Au (111) plane (**Figure** **2**a). OPE was utilized as a linker compound for primer conjugation onto the Au surface. The XRD spectrum of the deposited Au nanopillar layer showed a strong, sharp peak at 38°, indicating the (111) plane of the Au nanopillar surface.^[^ ^7^, ^26^, ^27^, ^28^ ^]^ XPS spectra were investigated to determine the binding energies of N 1s (398.75 eV) and S 2p (160.38 eV) to confirm the stability of the thiol‐ or OPE‐introduced Au nanopillar surface under thermal energy by the plasmonic effect (Figure 2b).^[^ ^29^, ^30^, ^31^, ^32^, ^33^ ^]^ Although peak intensities exhibited no significant change in the N 1s core level spectra, a decreased intensity was observed in the S 2p core level spectra. These results indicate the highly stable covalent bonding of OPE‐introduced Au nanopillar and were validated by theoretical demonstration through DFT simulations (Figure 2c).^[^ ^34^, ^35^ ^]^ The interaction energies were −2129.2, −2599.91, and −2979.12 kJ mol^−1^ for Thiol, NHC, and OPE, respectively, which were obtained by calculation following Equation (1).

(1) $$\Delta E_{\mathrm{Absolute}\;\mathrm{interaction}}=E_{\mathrm{binding}\;\mathrm{energy}}-\left[E_{\mathrm{gold}}+E_{\mathrm{interfacial}\;\mathrm{chemical}}\right]$$

Additionally, the orbital energy gap (OEG) of OPE‐introduced Au shows a smaller gap than NHC‐/thiol‐introduced Au.^[^ ^36^, ^37^ ^]^ Contact angle measurements were performed to observe the surface hydrophobicity properties, depending on the types of interfaces and thermal treatments (Figure 2d).^[^ ^38^ ^]^ The Au nanopillar had a 78.15 ± 2.6° angle, and the OPE‐ and thiol‐introduced nanopillars had 86.79 ± 2.66° and 86.55 ± 2.74°, respectively—an increase resulting from hydrophobicity due to the benzene rings in the inside group of each interfacial compound.^[^ ^39^ ^]^ Additionally, contact angle measurements were conducted after 40 thermal cycles (temperature range of 60—95 °C) for each interfacial compound to confirm thermal stability. The contact angle remained relatively stable (86.79°–84.41°) for the OPE‐introduced Au nanopillars, while it decreased markedly (86.55°–77.83°) for the SH‐introduced Au nanopillars. The decreased angle of the surface property observed in the SH‐introduced substrate resulted from the lower binding energies between thiol and Au compared with OPE. The unstable thiol bond toward thermal energy is illustrated in Figure 2c. Moreover, TOF‐SIMS was performed to confirm OPE incorporation onto the Au nanopillar surface; see the profile graph and 3D images shown in Figure 2e,f. The Au^−^, C^−^, and CN^−^ on the surface were investigated after OPE introduction. The C^−^ and CN^−^ exhibited a rapid reduction under 300 s sputter time.^[^ ^40^, ^41^ ^]^ The OPE layer formed onto the surface as a thin film was confirmed using sputtered ion profiles. In addition, the visualized elemental 3D images of Au^−^, C^−^, and CN^−^ showed intensities based on the sputtering time, revealing a high‐density thin layer formation from CN^−^ (Figure 2f).^[^ ^42^, ^43^ ^]^ To confirm the application of the OPE‐introduced Au nanopillar substrate in photonic PCR systems, thermocycling was measured under different light source exposures (Figure 2g). Thermocycling conducted at 60–95 °C for the general PCR reaction showed a similar consumption time relative to the pristine substrate shown in Figure 1g. These results indicate that the OPE interfacial compound does not influence energy conversion, as it maintains the consumption times of photon‐thermal conversion. Additionally, the isothermal property of the PCR system was measured to observe thermal maintenance during the gene denaturation steps, showing thermal accuracy within 0.1 °C (Figure S2, Supporting Information).

### 2.3 High‐Throughput Discrimination of Pathogens with a Plasmonic‐Based DNA Amplification Device

**Figure 3:**
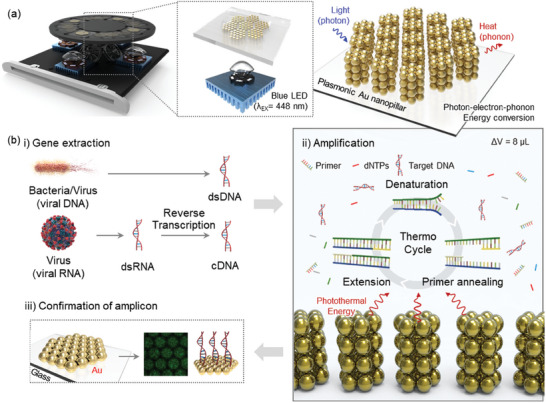
a) Schematic illustration of a fabricated high‐throughput platform with Au nanopillar arrayed plasmonic chip. b) The overall workflow of target‐dependent gene amplification reaction.

The plasmonic‐based gene amplification device features a light source, fluorescence detector, and amplification chamber as components (**Figure** **3**a). The energy of irradiated light to the Au nanopillar array is converted to thermal energy, which is utilized for gene amplification. As the light is irradiated to the bottom side of the plasmonic chip, the Au nanopillar array vertically provides a thermal gradient along the metal layer surface. Due to the induced thermal gradient, the solution contained in the chamber exhibited high velocity and superior heat transfer to the fluid. Compared to the flat metal layer, the Au nanopillar arrayed substrate possesses a higher surface‐to‐area ratio and shows remarkable photothermal conversion efficiency.^[^ ^12^ ^]^ The Au nanopillar array micropatterned into a honeycomb shape presents a more effective fluid flow that facilitates target gene diagnosis. Figure 3b shows the confirmation of various target diseases using the fabricated plasmonic‐based PCR device. The extracted genes from each disease (bacteria and virus) are treated differently. Depending on the type of gene, such as viral DNA or RNA‐containing disease, the PCR process varies. Although the DNA gene only needs DNA amplification, the RNA gene amplification process involves two major steps: reverse transcription and DNA amplification. The overall PCR protocol was configured accordingly. As presented in the illustration, at the amplification stage, thermal cycles were repeated 40 times between 95 and 55–60 °C. During those cycles, the PCR solution was mixed by turbulence effects in the adjacent area of the Au micropattern. At a denaturation temperature of 95 °C, the heated Au surface caused circulation of the PCR solution, resulting in a uniform directional flow. As the cooling fan was operated, the PCR solution was cooled down to 55–60 °C (the annealing process), presenting the flow of the solution from top to bottom. During the repetitive on/off light irradiation to the PCR chip substrate, the heated solution was at an ascending current, and the cooled down solution was at the descending current on the area of bare glass between Au patterns.

### 2.4 Confirmation of the Amplicon of Bacteria using Au Nanopillar Array

**Figure 4:**
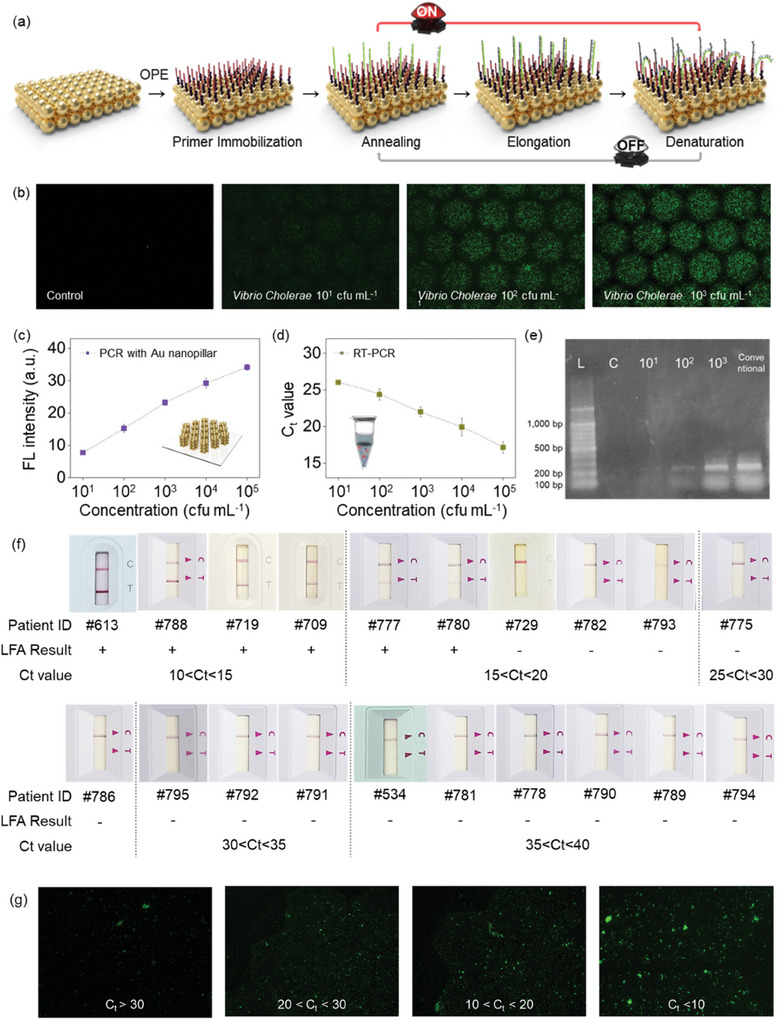
Results of an Au‐nanopillar arrayed plasmonic chip containing a gene amplification device. a) Schematic illustration of amplicon synthesis on the Au nanopillar array surface. b) Fluorescent image of the *Vibrio cholerae* gene‐amplified plasmonic chip surface showing variations corresponding to the concentration of the target amount. c) Standard curve of fluorescence intensity. d) Comparison of C_t_ value dependent on the amount of target pathogen and the error bars are given by the standard deviation. e) Agarose gel electrophoresis image after amplifying various amounts of target solution (L; DNA ladder, C; control w/o target DNA gene sample, 10^1^–10^3^; target solution with concentration variance). The diagnosis results of SARS‐CoV‐2 positive patients with the conventional diagnosis method f) lateral flow assay (LFA) and (g) photonic PCR (IRB approval number DGIRB 2021‐05‐003‐001).

For the rapid measurement of target diseases, fabricating miniaturized disposable devices is highly important. Previous studies used primer immobilization methodology to fabricate an on‐site gene amplification device.^[^ ^12^ ^]^ To demonstrate the use of the plasmonic‐based Au nanopillar substrate with PCR, all PCR reagents were loaded inside the PCR chamber, and the fabrication process is presented in Figure S3 (Supporting Information). The amplicon was synthesized through immobilized primers on the Au nanopillar array surface, and this synthesis was confirmed with fluorescence emission after the amplification procedure. The entire Au layer surface was treated with a thermostable chemical linker (OPE) (see **Figure** **4**a). The amine‐functionalized group was linked to the carboxylic group at the end of the primer. The primer was immobilized via the 5′ end‐functionalized primer through the C6‐amine group, and a free 3′ end was accessible for the annealing of complementary template DNA and DNA polymerase. On the primer‐immobilized Au nanopillar surface, the gene was continuously amplified during thermal cycles. The amount of one of the primers, either forward or reverse, was not abundant in the components of the PCR solution for gene amplification. The insufficient primer was immobilized on the Au nanopillar surface. The generated flow of the solution induced the attachment of the unreacted gene to the immobilized primer. The amount of amplicon was confirmed using green emission (SYBR) as the result confirmation methodology.

The versatility of our newly developed plasmonic chip is extendable to various diseases such as bacteria and viruses. For further investigation of the universal application of the Au nanopillar‐arrayed plasmonic substrate, we incorporated a highly selective primer set for bacteria. As the concentration of the bacterial gene increased, the intensity of fluorescence emitted from the Au nanopillar intensified, resulting in high amplification reaction kinetics proportional to the target gene concentration. The DNA gene solution, which was extracted from a serially diluted *Vibrio cholerae* solution, was applied to our novel Au nanopillar array platform (Figure 4b). The plasmonic‐based gene amplification system presented quantifiable signals with a linear range of bacteria amount of 10^1^–10^5^ cfu mL^−1^. The control sample lacking a DNA target gene showed no fluorescence over the entire Au surface. The plasmonic chip containing the gene 10^1^ cfu mL^−1^ showed faint green emission on the uniformly arrayed Au nanopillar, providing an LOD of 10^1^ cfu mL^−1^ in the solution. The fluorescence was confirmed at a *Vibrio cholerae* solution of 10^1^ cfu mL^−1^ (Figure 4c,d). In addition, to confirm the amplified results, the above solution of the Au nanopillar chip was analyzed with gel electrophoresis. The band was presented at the 250 bp size (Figure 4e). The amplification kinetics presented a linear correlation with target DNA in the PCR solution with a statistically significant signal difference. As shown in the image, each band intensity is *vibrio cholerae* concentration‐dependent. This characteristic value was comparable to the conventional RT‐PCR results. As shown in Figure 4e, the amplified results, presented as the emitting fluorescence intensity, were proportional to the C_t_ value obtained from standard RT‐qPCR. The experiment was triplicated using a DNA solution extracted from the *vibrio cholerae* solution. Additionally, to provide the validation results of Au nanopillar array integrated photonic PCR, we applied it to the genomic samples collected from COVID‐19 positive patients. The C_t_ values of the samples were confirmed with conventional RT‐PCR. As shown in Figure 4f,g, using the point‐of‐care tests (lateral flow assay; LFA) which is normally used as the gold standard for diagnosis, the sensitivity was compared to our newly developed methodology. Although the LFA showed the positive signal using the clinical sample under the C_t_ value of 15, the Au nanopillar array‐based diagnosis platform showed the positive signal with the clinical sample showing the C_t_ value over 30.

## 3 Conclusion

In this study, we demonstrated an advanced molecular diagnosis methodology with a plasmonic‐driven Au nanopillar arrayed gene amplification device. The fabricated Au nanopillar array showed superior thermal energy transfer efficiency to the PCR solutions due to the remarkable surface‐to‐volume ratio under light irradiation. The transferred photothermal energy from the Au nanopillar array facilitated a rapid thermal cycle (5.5 min per 40 cycles). The confirmation method for the amplicon on the Au surface was conducted directly using an ultrastable chemical linker. The thermostable PCR substrate was first developed by introducing an OPE interfacial chemical onto the Au nanopillar surface. Au nanopillars showed highly uniform structures, resulting in increased superficial area and binding density of OPE. The higher interaction energy (over two times) of OPE compared with thiol was proved by DFT simulation and calculation. Additionally, the OPE‐conjugated Au nanopillar substrate exhibited no different photon‐thermal conversion efficiency. Based on these results, our platform was validated with a genomic sample acquired from bacteria, showing rapid amplification and sensitive diagnosis performance. Our newly invented platform enables the early‐stage diagnosis and prevention of fatal pathogen dissemination.

## 4 Experimental Section

### Fabrication of Au‐Deposited Nanopillar Substrates

Here, an 8‐inch glass wafer was cleaned using a sulfuric acid hydrogen peroxide mixture solution. Au nanopillars were fabricated using microelectromechanical systems. The wafer was coated with a photoresist using a spin coater, and the micropatterns were created through a photolithography process using a micropatterned chrome mask and alignment (EVG6200, EVG). Au nanopillars (100–300 nm height) onto the micropatterned photoresist were deposited as Au by utilizing an E‐beam evaporator (KVET, KVT). The residual photoresist was removed through the lift‐off process using acetone and isopropyl alcohol. Finally, the dicing process progressed to obtain the separated substrates.

### Immobilization of Primers Via N‐Heterocyclic Carbene Compounds

Diisopropyl‐benzimidazolium hydrogen carbonate was synthesized through ion exchange under a reaction with hydrogen carbonate exchange resin (Amberlyst A26 hydroxide resin, Sigma–Aldrich). 10 g of resin reacted with 10 mL distilled water under bubbling with carbon dioxide for 0.5 h. The results were sonicated for 15 min, and the solution was filtered using a membrane filter (ADVANTEC, 0.4 µm, hydrophilic) to remove the precipitates. The purified solution was evaporated to remove the remaining water solution. 10 mL methanol was added to the powdered carbene compound. The final product was treated onto the Au nanopillar substrate and reacted for 12 h under an open environmental system. After covering the Au film with a carbene monolayer, the substrates were rinsed with methanol and dried under inert gas (N_2_ purge) 3 times. The final product was kept under vacuum conditions to maintain a stable state. pH 4.7 MES buffer solutions containing EDC (5.216 mM, Sigma–Aldrich) and sulfo‐NHS (2.763 mM, Sigma–Aldrich) were treated for 6 h to functionalize the terminal carboxyl group of the carbene compound. After removing the linker solution, a diluted 5 µM primer (Bioneer) solution was treated for over 6 h at 4 °C.

### Preparation of the Assembled PCR Chip

Onto the bare silicon wafer, a photoresist (DNR L300‐40) was coated and prebaked at 88 °C for 90 s. Using an aligner (MA‐6, SUSS MICROTEC), UV light was exposed under a circle pattern glass mask. The wafer was postbaked at 113 °C for 90 s and allowed to develop. With the fabricated wafer cast, a polydimethylsiloxane (PDMS) mold was fabricated by cast molding. A 10:1 w/w base and curing agent of a PDMS mixture (Sylgard 184 silicon elastomer kit) was prepared. The mixture was poured onto the wafer cast and cured in the 65 °C oven for 4 h. The solidified PDMS mold was detached from the cast and cut into independent pieces. At each side of the PDMS mold, the inlet and outlet ports were made using a punching tool. The functionalized Au nanopillar substrate and one side of the PDMS mold were treated with O_2_ plasma for 30 min. The PDMS mold and substrate were bonded after plasma treatment. The inner space of the PDMS mold was subjected to vacuum conditions to facilitate the injection of the PCR solution.

### Confirmation of the Amplicon on the Photonic PCR Chip

The fabricated PCR chip was loaded onto the stage of the portable PCR device. After the operation of the PCR process, the PCR chip was unloaded, and a 0.01X SYBR solution was treated onto the Au nanopillar surface. After 30 min incubation, the chip was washed, and the surface was analyzed using a fluorescence microscope.

### Instrumentations

AFM (NX20 300 & FX40) and UV–vis (Cary 5000 UV–vis–NIR) measurements were utilized to obtain the height profiles and absorption spectra. The photon‐thermal profiles were obtained using a homemade assembled device, and a thermal IR camera (FLIR E96) was used to capture the thermal changes. SEM (JSM‐7610F), XPS (K‐Alpha), and TOF‐SIMS (TOF.SIMS M6) were utilized to investigate the surface morphology and elemental analysis. DFT simulations and calculations for interaction comparisons were conducted using Gaussian 09 W and the Avogadro program.

### Ethical Approval

All procedures performed in research involving participants were in accordance with the ethical standards of the institutional and national research committee. Appropriate ethics committee approval and informed written consent of all participants were obtained.

## Conflict of Interest

The authors declare no conflict of interest.

## Supporting information



Supporting Information

## Data Availability

The data that support the findings of this study are available from the corresponding author upon reasonable request.
