# Supplementary material for: Plasmon‐Driven Gold Nanopillar Multiarrayed Gene Amplification Methodology for the High‐Throughput Discrimination of Pathogens
Source: Adv Sci (Weinh). 2025 Jan 14;12(9):2411849. doi: 10.1002/advs.202411849 (PMC11884620; doi:10.1002/advs.202411849)
Supplement: Supplementary file 1 — Supporting Information [file ADVS-12-2411849-s001.pdf]

ADVANCED SCIENCE
Open Access

Supporting Information

for *Adv. Sci.*, DOI 10.1002/advs.202411849

Plasmon-Driven Gold Nanopillar Multiarrayed Gene Amplification Methodology for the High-Throughput Discrimination of Pathogens

Sung Eun Seo, Kyung Ho Kim, Seo Jin Kim, Kyong-Cheol Ko, Woo-Keun Kim, Kyoung G. Lee* and Oh Seok Kwon**

Supporting Information

Plasmon-driven gold nanopillar multiarrayed gene amplification methodology for the high-throughput discrimination of pathogens

Sung Eun Seo,[a,†] *Kyung Ho Kim,*[a,†] *Seo Jin Kim,*[a] *Kyong-Cheol Ko,*[f] *Woo-Keun Kim,*[e,*]
Kyoung G. Lee,[b,*] *Oh Seok Kwon* [a,c,d,*]

Type	Target	Thermocycle (Cycles)	Time	Temperature range (°C)	LOD	Reference
Nanoislands-based Au nanopillar	SARS-CoV-2 and MERS-CoV standard DNA	30	3 min 30 s	60 ~ 98	0.1 ng/μL	[1]
TiN-based broadband meta-absorber	SARS-CoV-2 standard DNA	30	3 min 15 s	65 ~ 95	N/A	[2]
Au film	Commercial λ-DNA	30	< 5 min	55 ~ 95	N/A	[3]
Au nanorod	*S. aureus, S. epidermidis, E. coli*	30	13.8 min	55 ~ 85	10^2 CFU	[4]
Au nanofilm	SARS-CoV-2	40	475 s	60 ~ 94	10 copies/ μL	[5]
Au bipyramid nanoparticles	Bacteriophage M13	30	141.8 ± 12.4 s	72 ~ 95	1 pg/μL	[6]
Au nanofilm	pPICZ A vector template	30	< 13min	60 ~ 95	30 pg/μL	[7]
Au nanorod	*C. trachomatis*	30	9.5 min	60 ~ 85	1 copy	[8]
Au nanoshells	Commercial λ-DNA	40	800 s	60 ~ 91	50 pg	[9]
**Au nanopillar**	***Vibrio Cholerae* DNA**	**40**	**5.5 min**	**55 ~ 95**	**10^1 cfu mL^{-1}**	**This work**

Table S1. A comparison of previous research results of photonic PCR for the detection of pathogen.

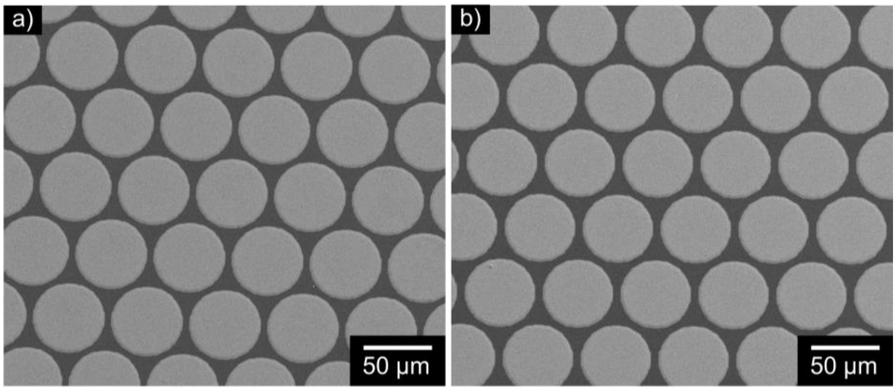

Figure S1. SEM images of the nanopillar structure a) before and b) after for the 40 thermal cycle exposure.

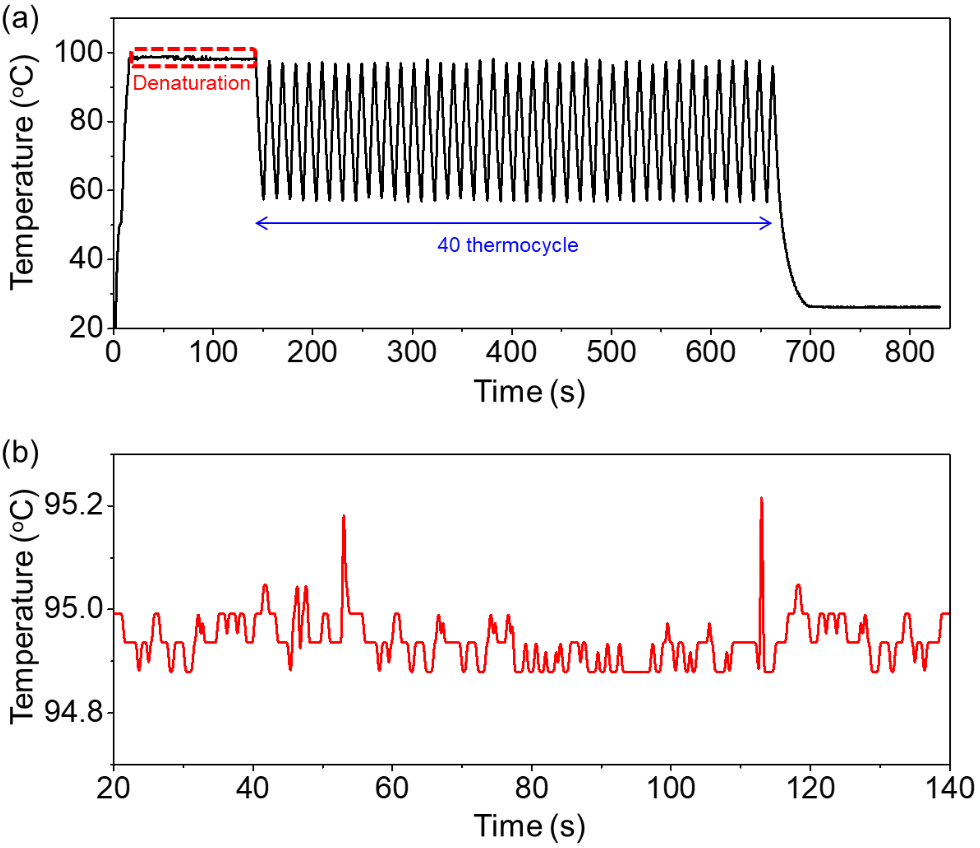

Figure S2. Thermal profile to investigate (a) the thermocycling consumption time and (b) the isothermal property.

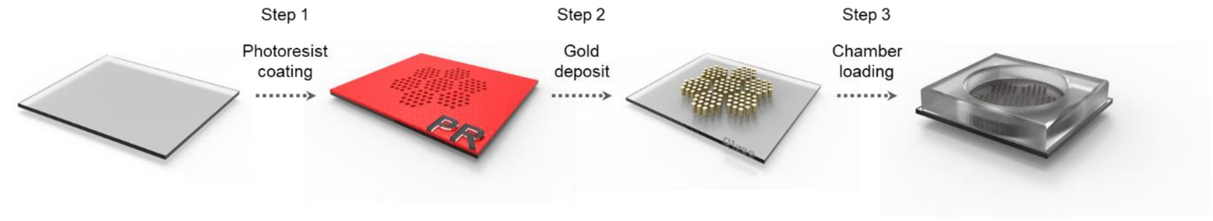

Figure S3. Fabrication process of gold nanopillar deposited plasmonic gene amplification chip.

Supporting Information References

[1] Y. Lee, B.-H. Kang, M. Kang, D. R. Chung, G.-S. Yi, L. P. Lee, K.-H. Jeong, *ACS Appl. Mater. Interfaces* **2020**, *12*, 12533.

[2] I. Kim, H. Kim, M. Go, S. Lee, D. Du Nguyen, S. Kim, K. Shrestha, A. Alsaadi, Y. Jeon, S. Jeong, G. Cho, J. K. Kim, J. Rho, L. P. Lee, *Adv. Mater.* **2024**, DOI 10.1002/adma.202311931.

[3] J. H. Son, B. Cho, S. Hong, S. H. Lee, O. Hoxha, A. J. Haack, L. P. Lee, *Light Sci. Appl.* **2015**, *4*, e280.

[4] J. Kim, H. Kim, J. H. Park, S. Jon, *Nanotheranostics* **2017**, *1*, 178.

[5] K. H. Kim, E. Ryu, Z. H. Khaleel, S. E. Seo, L. Kim, Y. H. Kim, H. G. Park, O. S. Kwon, *Biosens. Bioelectron.* **2024**, *246*, 115859.

[6] J.-H. Lee, Z. Cheglakov, J. Yi, T. M. Cronin, K. J. Gibson, B. Tian, Y. Weizmann, *J. Am. Chem. Soc.* **2017**, *139*, 8054.

[7] A. Jalili, M. Bagheri, A. Shamloo, A. H. Kazemipour Ashkezari, *Sci. Rep.* **2021**, *11*, 23338.

[8] P. Mohammadyousef, M. Paliouras, M. A. Trifiro, A. G. Kirk, *Analyst* **2021**, *146*, 5619.

[9] J. S. Ahn, W. Park, D. H. Jeong, S. B. Choi, S. H. Ahn, *Sci. Rep.* **2024**, *14*, 3895.
